# Suchtproblematik in der spezialisierten ambulanten Palliativversorgung in Deutschland: Ein Meinungsbild unter Leistungserbringern

**DOI:** 10.1007/s00482-025-00868-8

**Published:** 2025-02-13

**Authors:** Jannis Eersink, Julian Maul, Nils Heuser, Astrid Morin, Martin Gschnell, Christian Volberg

**Affiliations:** 1 Klinik für Anästhesie und Intensivtherapie, Universitätsklinikum Marburg, Philipps-Universität Marburg, Marburg, Deutschland; 2 Klinik für Anästhesiologie und Operative Intensivmedizin, Universitätsklinikum Köln, Köln, Deutschland; 3 Klinik für Dermatologie und Allergologie, Universitätsklinikum Marburg, Philipps-Universität Marburg, Marburg, Deutschland; 4 AG Ethik in der Medizin, Dekanat Humanmedizin, Philipps-Universität Marburg, Baldingerstraße, 35033 Marburg, Deutschland

**Keywords:** Palliativmedizin, SAPV, Medikamentenabhängigkeit, Substanzabusus, Suchterkrankung, Abhängigkeit, Palliative care, Specialised outpatient palliative care team, Drug abuse, Substance use disorder, Addiction

## Abstract

**Hintergrund:**

In der Palliativmedizin steht die Symptomkontrolle der Patienten im Vordergrund. Um dies zu erreichen, werden oft Medikamente eingesetzt, die ein Suchtpotenzial besitzen. Da Patienten in einer palliativen Erkrankungssituation immer länger überleben, kann es hierdurch zu einer Abhängigkeitsproblematik kommen.

**Ziele der Arbeit:**

Diese Studie untersucht, ob in der spezialisierten ambulanten Palliativversorgung (SAPV) Substanzmissbrauch bei Patienten als ein Problem wahrgenommen wird.

**Material und Methode:**

Eine Querschnittserhebung wurde unter allen deutschen SAPV-Diensten (*n* = 366) mithilfe eines Fragebogens durchgeführt. Die Daten sind deskriptiv ausgewertet worden.

**Ergebnisse:**

129 SAPV-Dienste (35,2 %) nahmen an der Umfrage teil. 49,6 % geben an, dass schätzungsweise 1–5 % ihrer Patienten an einer Medikamentenabhängigkeit leiden, und 65,9 % schätzen, dass 1–5 % ihrer Patienten Drogen konsumieren. 69,8 % der SAPV-Dienste screenen ihre Patienten nicht auf das Vorliegen einer Suchterkrankung, während dies von 3,1 % regelmäßig gemacht wird. Bei Vorliegen einer Suchtproblematik führen 65,9 % der SAPV-Dienste keine Maßnahmen durch.

**Diskussion:**

Laut den vorliegenden Daten wird Abhängigkeit nicht als Problem in der Palliativmedizin gesehen. Hier ist jedoch einzuschränken, dass fast kein SAPV-Dienst Patienten auf das Vorliegen einer Abhängigkeit screent, obwohl über die Hälfte schätzen, dass zumindest ein Teil der Patienten ein Suchtproblem aufweist. Hier ist in der Zukunft weitere Forschung notwendig, denn durch neue Therapien können Patienten in palliativen Situationen länger leben. Es wäre für die Weiterentwicklung der Palliativmedizin und die Lebensqualität der Betroffenen wichtig zu evaluieren, wie Patienten vor iatrogen induziertem Substanzmissbrauch geschützt werden können.

## Abstract (en)

**Background:**

In palliative care, the focus of treatment is on controlling the patient’s symptoms. To achieve this, drugs with addictive potential are often used. This can lead to addiction problems, as palliative care patients are surviving longer these days than they did a decade ago.

**Aims:**

This study investigates whether drug misuse and substance abuse are perceived as a problem in specialised outpatient palliative care teams.

**Materials and methods:**

A cross-sectional questionnaire survey was conducted among all German specialised outpatient palliative care teams (*n* = 366). Data were analysed descriptively.

**Results:**

A total of 129 palliative care teams (35.2%) participated in the survey. Of these, 49.6% stated that an estimated 1–5% of their patients suffer from medication dependency and 65.9% estimate that 1–5% of their patients have drug abuse issues. In all, 69.8% of palliative care teams do not screen their patients for the presence of addiction, while 3.1% do so regularly. If an addiction problem is present, 65.9% of the teams do not take any action.

**Conclusion:**

According to the available data, addiction is not considered a problem in German palliative care. However, it should be noted that almost no palliative care team screens patients for the presence of addiction, although more than half of the teams estimate that at least some patients have an addiction problem. Further research is needed here, as new therapies may help palliative care patients to live longer. It would be important for the development of palliative care and the quality of life of those affected to evaluate how to protect patients from iatrogenically induced substance abuse.

Palliativpatienten werden häufig mit potenziell abhängig machenden Medikamenten behandelt. Trotz Einzelfallberichten gibt es bis heute keine deutsche Studie, die sich mit der Thematik des iatrogenen Substanzabusus durch palliativmedizinische Symptomtherapien befasst. Dies wäre jedoch entscheidend, da auf der einen Seite Palliativpatienten oftmals analgetisch unterversorgt sind und viele Behandler und Patienten Angst vor Medikamentenabhängigkeiten haben, während auf der anderen Seite aber Palliativpatienten immer länger überleben und dadurch das Risiko für eine iatrogen induzierte Medikamentenabhängigkeit steigt.

## Einleitung

Die moderne Palliativmedizin ist eine relativ junge medizinische Spezialisierung, die sich erst um 1967 mit der Gründung des St. Christopher’s Hospice entwickelte [1]. Seitdem ist die Verbesserung der Lebensqualität bei Patienten, denen kurativ nicht mehr geholfen werden kann, das übergeordnete Ziel palliativmedizinischen Handelns, insbesondere durch eine gezielte Symptomkontrolle [2]. Hierbei steht besonders die Linderung von Schmerzen, Atemnot und Angst im Vordergrund [3–7]. Dies geschieht regelmäßig durch den Einsatz von Opioiden, Benzodiazepinen und anderen Medikamenten mit Abhängigkeitspotenzial, durchaus auch in hohen Dosierungen [8]. Jedoch ist bekannt, dass der Konsum größerer Mengen Opioide oder die längerfristige Einnahme von Benzodiazepinen die Gefahr der Abhängigkeit birgt [9].

Aber nicht nur die Palliativmedizin hat sich in den letzten Jahren weiterentwickelt, auch die Therapien in der Onkologie haben sich während des letzten Jahrzehnts grundlegend verändert. So konnte durch neue Therapieverfahren (z. B. Checkpointinhibition) die Überlebenszeit krebskranker Patienten in einer nicht mehr kurativen Situation deutlich verlängert werden [10]. Hieraus hat sich auch die Phase der Palliativbetreuung verlängert, da Patienten zum Teil „chronisch“ palliativ erkrankt sind. Die Kehrseite der Medaille ist jedoch, dass durch die genutzten Medikamente zur Symptomkontrolle, bei dafür vulnerablen Personen, die Gefahr der Substanzabhängigkeit entstehen kann. In der Literatur sind bereits verschiedene Beispiele, meist als Einzelfalldarstellungen, beschrieben [11–13], und auch in unserem klinischen Alltag haben wir auf der universitären Palliativstation in kurzer Zeit mehrere Patienten mit einer zum Teil iatrogen induzierten Medikamentenabhängigkeit betreut.

Medikamentenabhängigkeit kann, auch bei palliativen Patienten, zu einem deutlichen Verlust der Lebensqualität sowie großen Problemen in der weiteren Versorgung führen [14], auf der anderen Seite ist aber auch bekannt, dass ein relevanter Anteil der palliativ versorgten Patienten nicht ausreichend analgetisch behandelt ist [15]. So gibt eine schwedische Studie aus dem Jahr 2019 an, dass 25 % aller Patienten, die an Krebs oder einer chronischen Krankheit starben, in den letzten Lebenswochen an Schmerzen litten, obwohl 97 % von ihnen Opioide erhielten [16]. Hierfür gibt es verschiedene Vermutungen. Oft ist eine Befürchtung der Patienten jedoch, durch hohe Dosierungen von potenten Analgetika, insbesondere Opioiden, abhängig zu werden [17].

Diese Erhebung soll daher einen ersten Überblick verschaffen, inwieweit von Teams der spezialisierten ambulanten Palliativversorgung (SAPV) ein übermäßiger Medikamenten- oder Substanzkonsum bei palliativ betreuten Patienten in Deutschland wahrgenommen wird und ob dieser Konsum aus Sicht der behandelnden SAPV-Dienste ein Problem darstellt. Hierbei handelt es sich nach unserem Wissen um die erste Erhebung zu diesem Thema, die in Deutschland durchgeführt wird.

## Methode

Nach eingehender Literaturrecherche zur Thematik ist durch das Studienteam ein 23 Fragen umfassender Fragebogen erstellt worden. Mithilfe des Fragebogens soll in Erfahrung gebracht werden, inwieweit Sucht als Problem in der palliativmedizinischen Betreuung wahrgenommen wird, ob gezielt hierauf gescreent wird und ob gegebenenfalls Maßnahmen gegen eine Abhängigkeit ergriffen werden. Des Weiteren wird abgefragt, inwieweit die Gefahr einer potenziellen Abhängigkeit die Verschreibung von bestimmten Medikamenten beeinflusst.

Die Studie wurde nach positivem Ethikvotum der Ethikkommission Marburg (AZ: 139/22) im Deutschen Register klinischer Studien (DRKS-ID: DRKS00030427) registriert und der Fragebogen am 17. November 2022 zusammen mit einem Anschreiben und einem vorfrankierten Rücksendeumschlag an alle auf der Website der Deutschen Gesellschaft für Palliativmedizin (DGP) gelisteten SAPV-Dienste (*n* = 366) für Erwachsene und Kinder in Deutschland verschickt [18, 19]. Die Erhebung erfolgte anonymisiert, sodass kein Rückschluss auf die antwortende Organisation gezogen werden konnte.

20 Briefe konnten primär nicht zugestellt werden, woraufhin die Adressen der initialen Liste überprüft wurden und 19 der Briefe erneut versandt wurden.

Für die Auswertung wurden alle Antworten, die bis zum 30.04.2023 eingegangen waren, berücksichtigt. Die Daten wurden zumeist rein deskriptiv ausgewertet und für die Überprüfung von Gruppenunterschieden erfolgte die Signifikanztestung mithilfe einer einfaktoriellen ANOVA und dem Chi-Quadrat-Test. Freitextantworten wurden zunächst wörtlich übernommen und dann zu Themenfeldern zusammengefasst. Bei Angaben zu Medikamenten wurden diese in einheitliche Wirkstoffnamen und im Falle der Z‑Substanzen zu einer Wirkstoffklasse zusammengefasst. Für die Datenverarbeitung wurde Microsoft® Excel Version 16.68 genutzt.

## Ergebnisse

129 der 366 angeschriebenen SAPV-Dienste antworten über den Zeitraum von fünfeinhalb Monaten. Dies entspricht einer Rückläuferquote von 35,3 %. Hierbei sind 68,2 % der Fragebögen unter Mitwirkung des leitenden Arztes der SAPV und 27,9 % unter Mitwirkung der pflegerischen Leitung ausgefüllt worden. 86,8 % SAPV-Dienste behandeln ausschließlich Erwachsene. Die restlichen 13,2 % behandeln entweder ausschließlich Kinder oder sowohl Kinder als auch Erwachsene.

In einem ausgeglichenen Verhältnis sind die teilnehmenden SAPV-Dienste in einer Großstadt, Mittelstadt oder Kleinstadt bzw. im ländlichen Raum tätig. 13,2 % der SAPV-Dienste haben einen Arzt in ihrem Team, der über die Zusatzbezeichnung Suchtmedizinische Grundversorgung oder Suchtmedizin verfügt (vgl. Tab. 1). Es haben SAPV-Dienste aus allen deutschen Bundesländern an der Erhebung teilgenommen.

**Tab. 1:** Demografische Angaben der teilnehmenden SAPV-Dienste

	*n*	%
**Wer füllt den Fragebogen aus? (Mehrfachantwort)**		
*Kaufmännische Leitung*	10	7,8
*Zuständige(r)/leitende(r) Arzt/Ärztin*	88	68,2
*Pflegerische Leitung*	36	27,9
*Sonstige*	7	5,4
**Welche Wohnlage trifft auf Ihr Versorgungsgebiet zu? (Mehrfachantwort)**		
*Metropole (>* *1 Mio. EW)*	9	7,0
Großstadt (> 100.000 EW)	42	32,6
Mittelstadt (< 100.000 EW)	48	37,2
Kleinstadt, ländlicher Raum (< 20.000 EW)	41	31,8
Keine Angabe	3	2,3
**Wie viele Patienten betreuen Sie durchschnittlich pro Jahr?**		
*1–100*	13	10,2
*101–200*	20	15,7
*201–300*	27	21,3
*301–400*	17	13,4
*>* *400*	50	39,4
*Keine Angabe*	2	1,6
**Betreuen Sie Kinder oder Erwachsene?**		
*Erwachsene*	112	86,8
*Kinder*	10	7,8
*Beides*	7	5,4
**Haben Sie im Team Ärzte mit der Zusatzbezeichnung „Suchtmedizinische Grundversorgung“ oder „Suchtmedizin“**		
*Nein*	111	86,0
*Wenn ja, Anzahl:*	17	13,2
*1*	13	76,5
*2*	2	11,8
*3*	2	11,8

Knapp die Hälfte (49,6 %) der antwortenden Institutionen vermutet, dass 1–5 % ihrer Patienten an einer Medikamentenabhängigkeit leiden. Ein weiteres Viertel (26,4 %) schätzt, dass zwischen 6 und 10 % der betreuten Patienten eine Medikamentenabhängigkeit haben (vgl. Tab. 2).

**Tab. 2:** Vermuteter Anteil der betreuten Patienten mit Medikamenten- oder Drogenkonsum

	*n*	%
**Wie hoch schätzen Sie den Anteil Ihrer Patienten mit einer Medikamentenabhängigkeit ein?**		
*0* *%*	6	4,7
*1–5* *%*	64	49,6
*6–10* *%*	34	26,4
*11–20* *%*	15	11,6
*21–30* *%*	3	2,3
*31–100* *%*	2	1,6
*Keine Angabe*	5	3,9
**Bei wie viel Prozent dieser Patienten schätzen Sie diesen als iatrogen induziert ein?**		
*0*	10	7,8
*1–9* *%*	48	37,2
*10–25* *%*	24	18,6
*26–50* *%*	21	16,3
*51–75* *%*	9	7,0
*76–100* *%*	7	5,4
*Keine Angabe*	10	7,8
**Wie hoch schätzen Sie den Anteil Ihrer Patienten mit einem Drogenkonsum ein?**		
*0* *%*	37	28,7
*1–5* *%*	85	65,9
*6–10* *%*	4	3,1
*11–20* *%*	1	0,8
*21–30* *%*	0	0
*31–100* *%*	0	0
*Keine Angabe*	2	1,6
**Haben Sie den Eindruck, dass die Anzahl an Palliativpatienten mit einem Medikamenten- und/oder Drogenmissbrauch in den letzten 10 Jahren zugenommen hat?**		
*Ja*	14	10,9
*Nein*	84	65,1
*Bin mir unsicher*	30	23,3
*Keine Angabe*	1	0,8

**Abb. 1:**
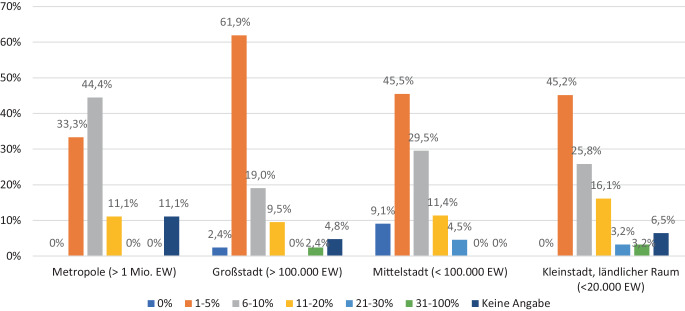
Geschätzte Medikamentenabhängigkeit in Bezug auf die Wohnlage

Bei der Frage, wie viele dieser Abhängigkeiten als iatrogen verursacht eingeschätzt werden, antworten 37,2 %, dass dies auf 0–9 % der Abhängigkeiten ihrer palliativ behandelten Patienten zutreffe (vgl. Tab. 2). Bei Vergleich der Antworten nach Wohnlage lassen sich hinsichtlich der geschätzten Abhängigkeit der betreuten Patienten keine Unterschiede erkennen (*p* = 0,45; vgl. Abb. 1).

Auch beim Vergleich der Antworten, ob es im Team der SAPV einen Arzt mit der Zusatzbezeichnung „Suchtmedizinische Grundversorgung“ bzw. „Suchtmedizin“ gibt, ergibt sich kein Unterschied zwischen den befragten Institutionen (*p* = 0,59). Auffällig ist jedoch, dass die Antwort, dass keiner der Patienten eine Medikamentenabhängigkeit habe, nie gewählt wurde, wenn es im Team einen Arzt mit entsprechender Zusatzbezeichnung gibt.

### Drogenkonsum

**Abb. 2:**
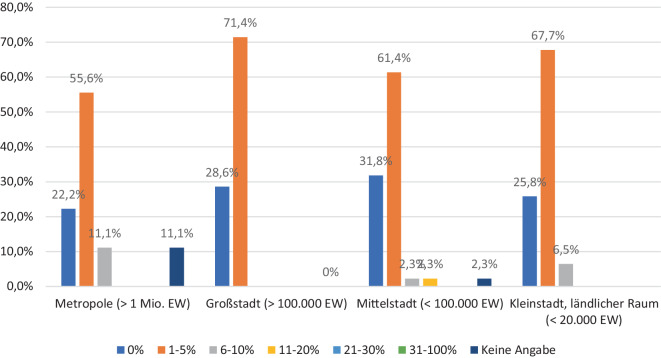
Geschätzter Anteil drogenabhängiger Palliativpatienten in Bezug auf die Wohnlage

Die Studienteilnehmer schätzen den Anteil ihrer Patienten mit einem illegalen Drogenkonsum als gering ein. So geben 28,7 % an, dass keiner ihrer Patienten Drogen konsumiere und 65,9 % schätzen, dass nicht mehr als 5 % ihrer Patienten illegale Drogen konsumieren (vgl. Tab. 2). Des Weiteren lässt sich kein nennenswerter Unterschied hinsichtlich eines potenziellen Drogenkonsums bei Patienten in Abhängigkeit von der Größe der Stadt bzw. Region, in welcher die SAPV tätig ist, feststellen (vgl. Abb. 2).

Auf die Frage, ob sich die Anzahl der Medikamenten- und Drogenabhängigen, welche durch einen SAPV-Dienst betreut werden, in den letzten 10 Jahren erhöht hat, antwortet die Mehrheit der Befragten mit „nein“ (65,1 %). Nur etwa jede zehnte Institution gibt an, dass sich die Anzahl der Abhängigen erhöht habe (vgl. Tab. 2). Hierbei ist jedoch zu beachten, dass 69,8 % aller SAPV-Dienste ihre Patienten überhaupt nicht auf das Vorliegen einer Drogen- oder Substanzabhängigkeit screenen. Nur 3,1 % der Befragten geben an, dies regelmäßig vorzunehmen, und 13,2 % bei Verdacht auf einen Substanzfehlgebrauch. Von den SAPV-Diensten, die ihre Patienten auf einen Substanzmissbrauch screenen, gibt der Großteil an, anstelle von validierten Erhebungsbögen „sonstige Methoden“ zu benutzen (60,5 %; vgl. Tab. 3). Bei der Frage, ob auch Angehörige auf einen Substanzmissbrauch gescreent werden, gibt die Mehrheit der SAPV-Dienste an, dies nicht zu tun (85,3 %). Lediglich 12,4 % geben an, Angehörige bei Verdacht auf einen potenziellen Substanzmissbrauch zu screenen.

**Tab. 3:** Screening und Einstellung zur Verordnung

	*n*	%
**Screenen Sie Ihre Patienten routinemäßig auf das Vorliegen eines kritischen Medikamenten- und/oder Drogenkonsums?**		
*Nein*	90	69,8
*Ja, aber nur bei Verdacht auf einen Substanzabusus*	17	13,2
*Ja, aber nur bei Aufnahme/Beginn der Betreuung*	11	8,5
*Regelmäßig*	4	3,1
*Keine Angabe*	7	5,4
**Wenn ja, welchen Screeningfragebogen benutzen Sie?**		
*ASSIST*	4	3,1
*DIPS*	2	1,6
*KFM*	12	9,3
*LBC*	1	0,8
*SKID-II*	1	0,8
*Sonstiges*	31	24,0
*Keine Angabe*	78	60,5
**Screenen Sie Angehörige auf das Vorliegen eines Substanzabusus?**		
*Ja*	0	0
*Ja, aber nur bei Verdacht auf einen Substanzabusus*	16	12,4
*Nein*	110	85,3
*Keine Angabe*	3	2,3
**Führen Sie bei Patienten mit einer Suchtproblematik Maßnahmen gegen die Abhängigkeit durch?**		
*Ja*	33	25,6
*Nein*	85	65,9
*Keine Angabe*	11	8,5
**Wie würden Sie die Einstellung im Team der SAPV zur Verordnung von Medikamenten mit Suchtpotenzial beschreiben?**		
*Sehr liberal*	17	13,2
*Liberal*	91	70,5
*Restriktiv*	16	12,4
*Sehr restriktiv*	0	0
*Keine Angabe*	5	3,9
**Inwieweit beeinflusst die potenzielle Gefahr einer möglichen Abhängigkeit Ihre Entscheidung zur Verordnung eines Medikaments?**		
*Sehr stark*	0	0
*Stark*	16	12,4
*Wenig*	71	55,0
*Sehr wenig*	38	29,5
*Keine Angabe*	4	3,1

Ebenfalls gibt die Mehrheit an, dass sie bei Patienten mit einer Suchtproblematik keine Maßnahmen gegen diese durchführt (65,9 %; vgl. Tab. 3). Lediglich ein Viertel führt Maßnahmen gegen eine Abhängigkeit durch. Im Freifeld antworten viele Studienteilnehmer, dass es sich aus ihrer Sicht aufgrund der nur noch begrenzten Lebenserwartung nicht lohne, hier Maßnahmen durchzuführen.

### Medikamente mit Abhängigkeitspotenzial

Die Studienteilnehmer wurden gefragt, welche Medikamente ihrer Meinung nach ein besonders hohes Abhängigkeitspotenzial haben. Hier werden zum Großteil Benzodiazepine (53,2 %) und Opioide (34,9 %) genannt. Andere Substanzen oder Substanzklassen sind nur in einem geringen Umfang angegeben worden (Pregabalin = 6,2 %, Cannabis = 5,4 %, Z‑Substanzen = 3,9 %).

### Einstellung zur Verordnung von Medikamenten mit Abhängigkeitspotenzial

Ein Großteil der Befragten bezeichnet die eigene Einstellung zur Verordnung von Medikamenten mit Abhängigkeitspotenzial als liberal (70,5 %) bis sehr liberal (13,2 %). Fast 85 % geben an, dass sie die potenzielle Gefahr einer Abhängigkeit wenig bis sehr wenig dahingehend beeinflusst, welche Medikamente sie verschreiben (vgl. Tab. 3). Zur Applikation von schnell wirksamen Opioiden werden vorwiegend bukkale (92,2 %), subkutane (89,9 %), nasale (68,2 %) oder intravenöse (58,1 %) Applikationsformen benutzt. Nur eine Minderheit nutzt rektale (14,0 %), inhalative (9,3 %) oder sonstige (13,2 %) Applikationsformen.

**Abb. 3:**
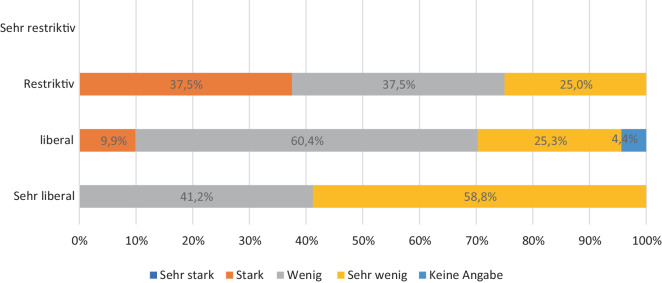
Verschreibungspraxis in Abhängigkeit von der Einstellung zu Medikamenten mit Abhängigkeitspotenzial

Wenn die Einstellung zur Verschreibung von Medikamenten mit einem Abhängigkeitspotenzial eher restriktiv ist, geben signifikant mehr Befragte an (*p* = 0,001), dass sie die potenzielle Gefahr einer abhängigkeitserzeugenden Wirkung des Medikaments in ihrer Entscheidung zur Verordnung stark beeinflusst (37,5 %; vgl. Abb. 3).

### Beeinflussung beim Verschreiben in Abhängigkeit von der Einstellung des Verschreibenden

Bei fast allen SAPV-Diensten sind die jeweiligen SAPV-Ärzte verantwortlich, wenn es um die Verschreibung potenziell abhängig machender Medikamente geht (96,1 %). 43,4 % geben darüber hinaus an, dass die Verordnung zusätzlich auch durch Hausärzte erfolgt. Von 14,7 % wird angegeben, dass ebenfalls Schmerztherapeuten entsprechende Medikamente verordnen.

91,5 % aller Befragten geben an, dass dokumentiert wird, wie viele und welche Medikamente genau der Patient einnimmt. Hingegen dokumentieren 6,2 % dies nicht.

Insgesamt wünschen sich 62 % der Teilnehmer mehr Fortbildungsangebote zum Thema Sucht in der Palliativmedizin, während 34,9 % dies nicht wünschen.

### Sonstige Anregungen

In Freitextantworten haben mehrere Studienteilnehmer darauf hingewiesen, dass nicht die Abhängigkeit, sondern eine Unterversorgung mit Schmerzmitteln ein großes Problem der Patienten sei. Hierbei ist auch immer wieder genannt worden, dass dies oftmals aus Angst der Patienten vor einer potenziellen Abhängigkeit geschehe. Viele SAPV-Dienste verweisen auch darauf, dass sie Patienten nur über eine kurze Zeitspanne betreuen und Suchtprobleme daher unerheblich seien.

## Diskussion

Bei Betrachtung der Ergebnisse zeigt sich, dass Medikamenten- oder Substanzabhängigkeit aus Sicht der befragten SAPV-Dienste nur ein geringes Problem palliativ betreuter Patienten in Deutschland zu sein scheint. Jedoch ist einschränkend zu erwähnen, dass auch nur wenige SAPV-Dienste ihre Patienten auf einen problematischen Medikamenten- oder Drogenkonsum hin screenen.

In der Literatur finden sich nur wenige Studien, die sich dem von uns untersuchten Themenfeld widmen. Häufig werden Studien zum Thema Medikamentenabhängigkeit an Patienten mit chronischem Schmerz durchgeführt, welche jedoch nicht auf Palliativpatienten übertragen werden können.

Eine bereits ältere Publikation aus 2007 hat sich dem Thema Sucht und Substanzmissbrauch bei palliativmedizinisch betreuten Patienten gewidmet [20]. Diese Studie beschreibt, dass bis zu 7,7 % aller Krebspatienten an einer Medikamentenabhängigkeit leiden, ohne dass hierbei jedoch genau zwischen Krebs- und Palliativpatienten unterschieden wird [20]. Gerade die Patienten, die durch einen SAPV-Dienst betreut werden, leiden oft bereits länger an einer Tumorerkrankung und werden dementsprechend länger mit Analgetika behandelt. Ebenso sind es gerade die durch einen SAPV-Dienst betreuten Patienten, welche eine komplexe und schwer einzustellende Schmerzproblematik vorweisen [21]. So ist es nicht verwunderlich, dass es bei Palliativpatienten zu einer Zunahme der Anzahl der verschriebenen Medikamente kommt, obwohl die Anzahl der Medikamente, die für komorbide Erkrankung eingenommen werden, sinkt. Die beschriebene Zunahme entsteht hierbei durch vermehrte Verschreibung von Medikamenten zur Symptomkontrolle [22]. Dies trifft auf eine nicht zu unterschätzende Patientenanzahl zu, die in der letzten Lebensphase eine spezialisierte palliativmedizinische Versorgung benötigen [2]. Ein Artikel von Bruera und Paice stellt ebenfalls heraus, dass onkologisch betreute Patienten oft große Mengen opioidhaltiger Analgetika einnehmen, da andere Therapieoptionen, wie zum Beispiel Physiotherapie, nicht ausreichend genutzt werden. Nach Meinung der Autoren sollten sich Behandler immer die Frage stellen, warum hohe Dosen Opioide eingenommen werden. Dies kann auf eine tatsächliche Abhängigkeit zurückzuführen sein, oder um andere Probleme, wie Ängste oder Trauer, zu lindern [23].

Im Widerspruch zu den von uns erhobenen Zahlen steht eine Umfrage unter Palliativmedizinern aus den USA aus dem Jahr 2012. Sie ergab, dass 77,2 % der Ärzte in den letzten zwei Wochen einen Patienten mit einer Substanzgebrauchsstörung betreut haben. Außerdem gaben 43,9 % der befragten Palliativmediziner an, in den letzten zwei Wochen einen Patienten mit einem Opioidmissbrauch behandelt zu haben [24]. Eine weitere Studie aus dem Jahr 2015 bestätigt diese Einschätzung. So wurden über 6 Monate alle Patienten, die in einer onkologischen Klinik in den USA betreut wurden, auf Abhängigkeiten untersucht. Dabei ergab die Erhebung, dass 46 % der Patienten einen kritischen Screening-Wert aufwiesen [25]. Auch die INTERREG-Studie aus Deutschland stellt dar, dass rund 30 % der stationären Patienten im Krankenhaus einen übermäßigen Alkoholkonsum betreiben und bei einem Drittel der untersuchten Seniorenheimbewohner Benzodiazepine nachgewiesen werden konnten. Das zugrunde liegende Suchtverhalten ist aber den Behandlern zumeist nicht bekannt [26].

71 % der befragten Palliativmediziner einer anderen amerikanischen Studie berichten, dass sie Urintests nutzen, um ihre Patienten auf einen Substanzmissbrauch zu screenen [27]. In unserer Studie gibt die Mehrheit der Befragten an, ihre Patienten gar nicht auf einen Substanzmissbrauch zu untersuchen. Nur 3,1 % geben an, regelmäßig zu screenen, wovon jedoch niemand angegeben hat, dies mit einem Urintest durchzuführen. Es ist somit nicht ausgeschlossen, dass der Anteil der Patienten mit einem Substanzmissbrauch höher ist als von den Behandlern angenommen. Es muss also davon ausgegangen werden, dass zurzeit in Deutschland die Anzahl der Palliativpatienten, die von einer SAPV betreut werden und an einer Substanzabhängigkeit leiden, nicht bekannt ist. Dies wäre jedoch für die Versorgung der Patienten wichtig, da gerade eine neuentstandene Suchtproblematik auch in der palliativen Erkrankungsphase negative Auswirkungen auf die Betroffenen haben kann.

Auf der anderen Seite haben viele Patienten Angst vor der Einnahme starker Schmerzmittel, insbesondere vor Opioiden, da sie befürchten, von diesen abhängig zu werden. Dies lässt sich auch aus den Freitextantworten erkennen, in denen mitgeteilt wurde, dass Patienten aus Angst vor einer Abhängigkeit nicht ausreichend analgetisch behandelt werden. Dies gibt auch Himstedt-Kämpfer als einen der Gründe für eine unzureichende Symptomkontrolle an [17]. Mit konkreten Zahlen über die Anzahl palliativmedizinisch betreuter Patienten mit einer Abhängigkeit könnten Patienten erstmalig angemessen über das Nutzen-Risiko-Verhältnis einer adäquaten Schmerztherapie aufgeklärt werden. Dies ist insbesondere wichtig, da viele Patienten, die an Malignomen oder einer chronischen Krankheit sterben, in den letzten Lebenswochen an Schmerzen leiden, obwohl 97 % von ihnen Opioide erhalten [16].

Palliativmediziner sollten im Erkennen, Vorbeugen und Behandeln von Medikamenten- und auch Substanzabhängigkeit geschult werden und diese Aufgaben in ihrer Arbeit berücksichtigen. Jedoch sehen sich viele Palliativmediziner selbst nicht als Experte für diese Themen [28]. Sie sind aber oft die letzten Ärzte, die die Möglichkeit haben, Abhängigkeitserkrankungen zu therapieren [29], und werden auch von anderen Medizinern als Experten hierfür gesehen [28]. Eine Behandlung der Suchterkrankung erhöht die Lebensqualität der betroffenen Patienten [30]. Diese Möglichkeit zur Verbesserung der Lebensqualität sollte auch palliativmedizinisch betreuten Patienten nicht verwehrt werden. Jedoch sollte hierbei stets der individuelle Patientenwunsch und ggf. Leidensdruck im Vordergrund stehen. Aufgrund der Besonderheiten palliativmedizinisch betreuter Patienten sollten hierbei auch immer die Krankheits- und Abhängigkeitsgeschichte, die Lebenserwartung sowie die persönlichen Ressourcen des Patienten mitberücksichtigt werden. Bei langjährig suchtkranken Patienten oder Personen mit kurzer Lebenserwartung steht die Behandlung der Sucht sicher nicht im Vordergrund, hingegen sollte bei Patienten mit erst kurzfristig entstandenem Abusus oder längerer Lebenserwartung das Potenzial eines Entzugs bedacht werden.

## Limitationen

Diese Studie kann nur einen ersten Überblick über die Abhängigkeitsproblematik ambulant betreuter Palliativpatienten geben. Besonders das Studiendesign, in dem die Behandler selbst den Anteil medikamenten- und drogenabhängiger Patienten schätzen, schwächt die Aussagekraft der vorliegenden Daten. Hier wären weitere Untersuchungen mit objektiv erhobenen Daten (z. B. durch Screening) notwendig, um konkrete Aussagen machen zu können. Auch kann die Abgabe sozial erwünschter Antworten zu einer Verzerrung der Ergebnisse geführt haben.

## Schlussfolgerung

Palliativmediziner sollten sich mit Fragen des Substanzmissbrauchs ihrer Patienten befassen, da Patienten immer länger in palliativmedizinischer Behandlung bleiben. Hierdurch steigt bei dafür vulnerablen Personen das Risiko eines Substanzabusus. Da Abhängigkeitsproblematiken den Behandlern oft nicht bekannt sind, wäre ein großzügigeres Screening sinnvoll, um einen besseren Überblick über das Nutzen-Risiko-Verhältnis der eigenen Behandlung zu erlangen. Auch Aus‑, Fort- und Weiterbildungen zu diesem Thema sind notwendig, um Mitarbeitende hierfür zu sensibilisieren. Ärzte wären so besser in der Lage, auf Risiken zu achten und gleichzeitig das ganze Potenzial einer adäquaten Symptomkontrolle für ihre Patienten zu nutzen.

## Data Availability

Die Daten können mit speziellem Anlass beim korrespondierenden Autor angefragt werden.
